# Rethinking the bioavailability and cellular transport properties of S-adenosylmethionine

**DOI:** 10.15698/cst2022.01.261

**Published:** 2021-12-06

**Authors:** Yudong Sun, Jason W. Locasale

**Affiliations:** 1 Department of Biochemistry, Duke University School of Medicine, Durham, North Carolina USA 27710.; 2 Department of Pharmacology and Cancer Biology, Duke University School of Medicine, Durham, North Carolina USA 27710.

**Keywords:** S-adenosyl-methionine, SAM, methionine, metabolism

## Abstract

S-adenosylmethionine (SAM) is a versatile metabolite that participates in a wide range of reactions such as methylation and transsulfuration. These capabilities allow SAM to influence cellular processes such as gene expression and redox balancing. The importance of SAM is highlighted by its widespread usage as an over-the-counter nutrient supplement and as an experimental reagent in molecular biology. The bioavailability and cellular transport properties of SAM, however, are often overlooked under these contexts, putting limits on SAM's therapeutic potential and complicating the interpretation of experimental results. In this article, we examined the chemical stability and cellular permeability of SAM, proposed a schematic for indirect SAM transport across the mammalian plasma membrane, and lastly discussed the implications arising from such transport schematic.

## INTRODUCTION

As one of the rare sulfonium metabolites present in eukaryotic cells, S-adenosylmethionine (SAM) is remarkably versatile. Owing to the electron-deficient sulfur inside SAM, the covalent bond between the sulfur and its neighboring groups are uniquely susceptible to nucleophilic attack. This property allows the transfer of these neighboring groups onto other molecules, and enables SAM to participate in a diverse set of chemical reactions including methylation, transsulfuration, and aminopropylation amongst others [1]. Cells employ these reactions to perform tasks such as protein and nucleic acid methylation, sulfur amino acid metabolism and polyamine synthesis, and, in turn, to orchestrate gene expression, redox status and other crucial processes. Given the ubiquity of these SAM-dependent processes, alternation of intracellular SAM availability thus could elicit profound impacts on cellular biology, physiology, and ultimately diseases.

The importance of SAM is also reflected by the widespread public interest in using it as an over-the-counter nutrient supplement with purported effects as a therapeutic agent to ameliorate conditions such as liver diseases and depression. At the same time, SAM is also frequently utilized in tissue culture experiments as a reagent to elucidate the functions of methionine metabolism and putative SAM-related processes. It is worth noting, however, both the cellular availability and the mode of action of supplemented SAM are unclear. This could lead to important implications in its usage both as a therapeutic drug and a reagent in molecular biology experiments. Here we discuss this issue in detail.

## SAM AVAILABILITY AND LIVER DISEASES

Multiple studies have observed decreased hepatic SAM biosynthesis in different forms of chronic liver injury [2]. This SAM deficiency often co-occurs with impaired hepatic methionine metabolism and reduced activity of the SAM synthase (MAT) [2], which condenses methionine and adenosine triphosphate (ATP) to form SAM. On the other hand, SAM supplementation in animal models has been demonstrated to alleviate alcohol-induced liver damage, and to improve survival rate in drug-induced hepatotoxicity and liver injuries [3]. In addition, studies in humans have suggested that the ingestion of SAM is generally well tolerated with an excellent safety profile [4]. These results have prompted interests in using SAM as a therapeutic agent for human patients with liver injuries. Meta-analyses of early small-scale randomized clinical trials have found significant results in reducing mortality [2]. However, studies have also suggested that orally administered SAM has a rather poor bioavailability, with the area under the plasma concentration-time curve (AUC) ranging from 0.58% to 1.04% of the AUC of intravenously administered SAM [5]. For additional context, the SAM concentration at various organ tissue has a reported range of 3.5-9 nmol/100 mg tissue [6] and the plasma SAM concentration has been reported in the range of 50-150 nmol/L [7, 8]. Studies performed in human volunteers have found no significant increases in blood SAM concentration when an oral dose of 10 mg/kg was administered [9]. Experiments involving radioactively labeled SAM have suggested that the carbon, hydrogen, and sulfur of SAM can be effectively incorporated into the body even with oral administration [4]. However, it is unclear whether such incorporation occurred through intact SAM or through its degradation products. With SAM's mode of action remaining poorly understood and the low bioavailability of SAM with oral supplementation, its therapeutic potential is complicated.

## SAM AS A REAGENT IN TISSUE CULTURE EXPERIMENTS

As the immediate catabolic product of the essential amino acid methionine, SAM is positioned in the nexus of nutrient sensing networks and it exerts influences over important cellular decisions such as proliferation, differentiation, and autophagy in response to the dynamic nutrient environment [10]. Consequently, the interplay of SAM and these processes has been heavily investigated by researchers in a host of studies [11]. Common to many of these studies, the initial identification of SAM-related genes is often followed up by complementation or rescue experiments with direct SAM supplementation to further test the causation link between SAM availability and observed phenotypes. In these experiments, SAM is often directly added to the cell culture medium over the duration of the experiments, which could range from overnight to a couple of days, such as in the case of cell proliferation. The cellular availability of such forms of SAM supplementation, however, is rarely discussed and could have implications in interpreting the experimental results.

## SAM AVAILABILITY IN ANIMAL EXPERIMENTS

In contrast to tissue culture experiments, where the components of the culture medium are to some extent well defined, animal experiments commonly involve diet with less well-defined chemical compositions compounding on top of the interactions between microbiota, food, and the host. These additional factors might all influence the availability of a supplemented nutrient to the animal in studies. For example, the enzyme L-methioninase, which degrades methionine, is ubiquitous in fungi and bacteria [12]. Given that SAM is the immediate metabolic product of methionine, it is likely that SAM availability and metabolism could also be influenced by the methionine metabolism of the host microbiota. Besides potentially acting through methionine, the microbiota could also influence the host's SAM metabolism through B-vitamin production and choline consumption [13]. Some of the added complexities from the host-microbiota interactions can be studied through manipulations of metabolic genes that control the utilization of a given nutrient. For most of the cases, however, such isolation remains challenging, and the impact of host-microbiota interactions in general remains poorly understood. Thus, this article will primarily focus on SAM transport and availability at the cellular level.

## TRANSPORT PROPERTIES OF SAM

**Figure 1:**
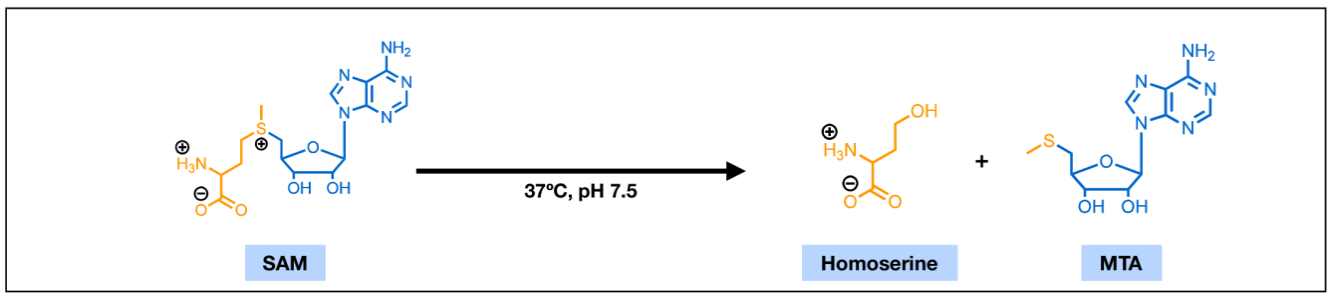
FIGURE 1: The chemical structures of S-adenosylmethionine (SAM) and 5'-methylthioadenosine (MTA). With features of an amino acid (yellow) and a ribose nucleotide (blue), SAM is highly polar. Under physiological conditions, SAM can undergo a non-enzymatic cleavage reaction and degrade into homoserine and MTA.

Synthesized from methionine and ATP, SAM is highly polar (**Figure 1**). This polar nature presents challenges for its passive diffusion across biological membranes. Cellular uptake studies of SAM in hepatocytes have revealed a low level of cellular accumulation with a ratio of intracellular to extracellular SAM concentration of 0.19 μM : 1 μM at equilibrium [14]. Additional studies performed in an intestinal epithelial model have reported that the apparent permeability coefficient of SAM (0.6×10^-6^ to 0.7×10^-6^ cm/s) is much lower than the typical value for passive diffusion [14], and suggested that the main mode of SAM transport is paracellular transport, by which molecules primarily travel through the tight junction. These findings are echoed by the likely absence of SAM transporters in the plasma membrane of mammalian cells. Past studies have successfully identified SAM transporters in the plasma membrane of yeast (SAM3) [15], and in the inner membranes of human mitochondria (SAMC) [16]. However, the lack of mammalian SAM3 orthologue, and the mitochondrial-localization of SAMC [16] suggest the lack of a dedicated SAM transporter in the mammalian plasma membrane and further complicate the uptake of SAM from the extracellular environment. These findings might lead one to reason that the direct SAM supplementation may render little to no influence over cellular biology since it cannot be efficiently absorbed by cells, yet successes have been observed in clinical trials and in laboratory experiments. The paradox, however, could potentially be resolved if SAM enters the cell, not in its intact form, but in the forms of its breakdown products, and then reassembles back to SAM inside the cell.

## CHEMICAL STABILITY OF SAM UNDER PHYSIOLOGICAL CONDITIONS

Common cell culture media are usually buffered to a slightly basic condition. A solution environment with such pH and a temperature of 37°C has been demonstrated detrimental to SAM's covalent stability. Liquid chromatographic studies suggested that SAM is markedly unstable at pH 7.5 and can rapidly degrade into 5'-methylthioadenosine (MTA) through a non-enzymatic cleavage reaction [17] (**Figure 1**). Efforts towards making more stable salts of SAM, such as the widely used sulfate and *p*-toluenesulfonate double salts of SAM, have yielded improved dry-state stability and greatly extended its shelf-life [18]. The in-solution stability, however, remains mostly unaddressed. The half-time of MTA formation from in-solution SAM has been reported ranging from 16 to 42 hours [1]. This range of half-time overlaps with the length of many rescue experiments in tissue culture systems and could have major implications in the intracellular SAM availability over the course of these experiments.

## CELLULAR PERMEABILITY OF 5'-METHYLTHIO-ADENOSINE (MTA) AND THE METHIONIN E SALVAGE PATHWAY

Unlike SAM, MTA can readily cross the plasma membrane of mammalian cells [19, 20]. With a chemical structure similar to that of an adenosine nucleoside (**Figure 1**), MTA can enter the cell through the nonspecific nucleoside transport system [19]. Additionally, kinetics studies have suggested that MTA might also enter the cell through passive diffusion, which could account for over 50% of its influx in certain cases [19]. This opens the possibility for cells to utilize extracellular MTA from degraded extracellular SAM.

**Figure 2:**
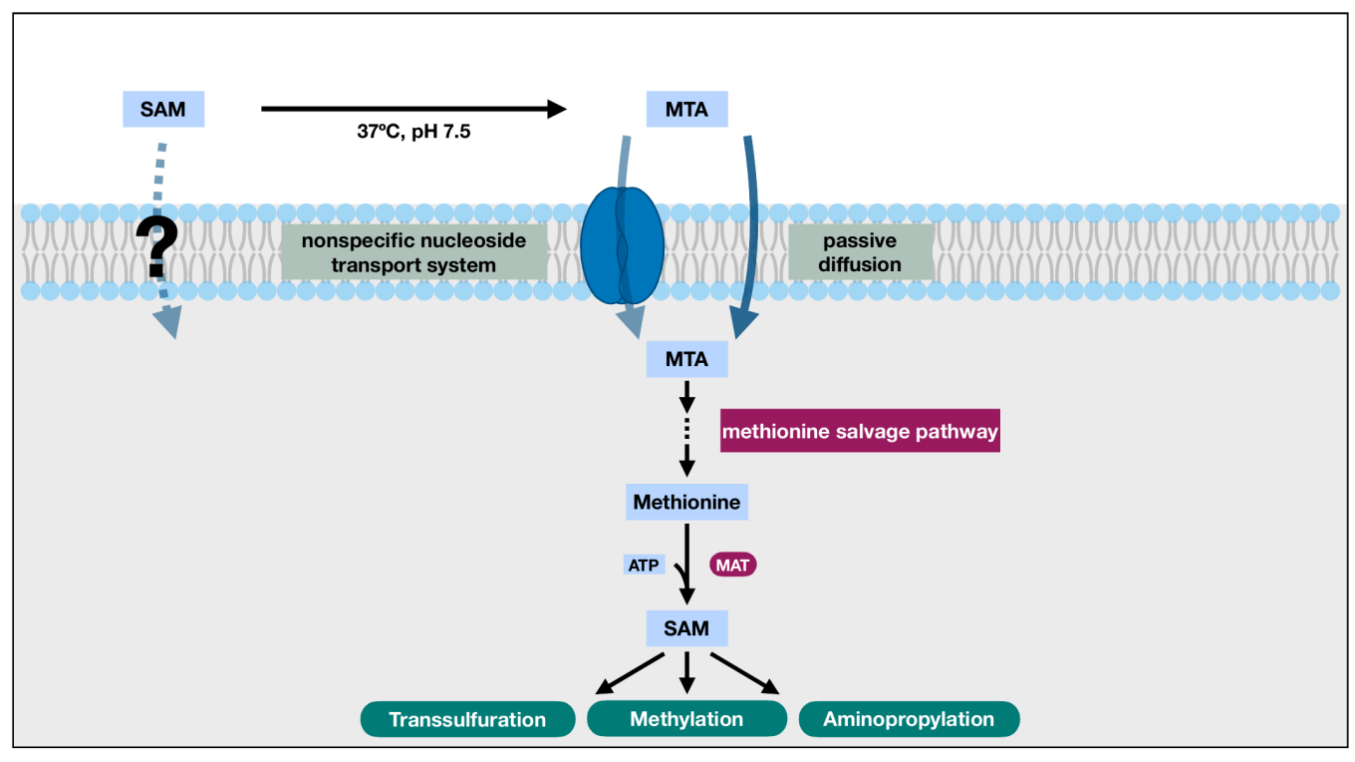
FIGURE 2: The indirect SAM transport schematic. The polar nature of SAM and the likely absence of SAM transporters in the plasma membrane presents challenges for the direct transport of SAM across the plasma membrane and limits its bioavailability. Under physiological conditions, however, SAM readily degrades into MTA. Unlike SAM, MTA can cross cell membrane through both nonspecific nucleoside transport system and passive diffusion. Once inside the cell, MTA can participate in the methionine salvage pathway to regenerate methionine, which can be utilized by the SAM synthase, methionine adenosyltransferase (MAT), to replenish intracellular SAM. When viewed as a whole, MTA effectively brings in the sulfur and activated methyl group of the extracellular SAM into the cell, overcomes the challenges of direct SAM transport across plasma membrane, and enables the utilization of those functional groups for reactions such as methylation and transsulfuration.

Normally produced as a byproduct during polyamine synthesis, MTA is often recycled into methionine through a series of enzymatic reactions collectively known as the methionine salvage pathway. In this pathway, MTA's methylthio group and the carbon backbone of its ribose are retained and eventually transformed into methionine [21]. The methionine generated through this process can then be combined with cellular ATP to replenish intracellular SAM. Crucially, it has been observed that the methionine salvage pathway can be co-regulated with certain SAM demanding processes, such as polyamine synthesis in yeast [21], potentially to help maintain SAM availability. Such observations suggest the possibility of an indirect transport mechanism through the sulfur and the methyl group of extracellular SAM using MTA as the carrier (**Figure 2**). In fact, studies have demonstrated that the passive diffusion of MTA alone can support a methionine salvaging capacity of at least 50 μM, and the supplementation of MTA can support the short-term growth of lymphoblasts on a methionine depleted cell culture medium [22].

This indirect SAM transport schematic, capable of explaining the rescue effect of extracellular SAM in tissue culture systems, however, has its limitations in restoring the decreased intracellular SAM levels observed in liver disease models. In many of these models, the SAM synthase, MAT, is often found defective or transcriptionally inhibited [23]. Consequently, the salvaged methionine from MTA might not be able to restore the lowered intracellular SAM availability. However, a study has reported that MTA can mimic SAM's inhibition on TNF-α expression in certain systems [24], which might partially underlie SAM's anti-inflammatory and hepatoprotective effects, thus providing a viable alternative mode of action for this schematic.

Together, given the covalent instability and membrane-impermeable nature of SAM, and MTA's capability of crossing the cell membrane, participating in methionine salvaging, and eliciting anti-inflammatory effects, it is possible that the observed effects of extracellularly supplemented SAM in tissue culture experiments and in clinical usage are achieved through the salvaged methionine and SAM from MTA or through MTA itself.

## IMPLICATIONS OF THE DIRECT SAM TRANSPORT SCHEMATIC

If SAM supplementation exerts its effects through MTA, several implications could arise. First, in addition to providing methionine and SAM to the cell, this schematic could also lead to an increase in MTA levels that are normally not present during normal SAM metabolism. MTA itself can influence various cellular processes. For example, studies have reported that MTA possesses inhibitory effects over histone methylation and it was also reported that MTA could inhibit the activity of S-adenosylhomocysteine (SAH) hydrolase [1]. The latter could lead to the buildup of SAH, which is a potent inhibitor of many methyltransferases and is closely intertwined with one-carbon metabolism through the methionine cycle [25]. Indeed, cellular toxicity of MTA has been observed in lymphoblasts when the concentration of supplemented MTA exceeds 50 μM [22]. Second, the methionine salvage pathway underpinning this indirect SAM transport schematic is highly delicate. With at least five enzymatic reactions in between MTA and methionine [21], defects of any one of the enzymes could render the pathway broken. For instance, the methylthioadenosine phosphorylase (MTAP), which catalyzes the first step in MTA's conversion into methionine, is commonly found defective in many cancer cell lines [25]. Such defects could complicate the result interpretation of experiments involving SAM supplementation. Third, due to the presence of the methionine salvage step, supplementation of SAM could also lead to increased intracellular methionine availability. This presents a challenge when one wants to parse the other roles of methionine, such as protein synthesis and nutrient sensing, from its role in methyl group donation via SAM**.** Last, on the therapeutic side, considerable amounts of effort have been directed towards the development of SAM salts/analogues with longer shelf-life while leaving the aspects of cellular permeability largely unaddressed. Such approaches, on the contrary, could potentially further limit the therapeutic efficacy of SAM, due to less degradation of SAM into MTA for methionine salvaging and SAM synthesis inside the cell.

## FUTURE DIRECTIONS

Given the caveats of extracellular SAM supplementation and the implications of the indirect SAM transport schematic, one might want to consider the following points when designing experiments involving SAM supplementation or improving SAM as a therapeutic drug. First, the genetic background of the experiment system should be examined, especially regarding genes that are involved in the methionine salvage pathways. Second, one might consider performing MTA supplementation in parallel to SAM supplementation to further strengthen the causation link between SAM and the observed phenotype. Third, genetic manipulations or pharmacological modulation of SAM utilizing enzymes might be helpful to discern between the contribution of SAM and the contribution of methionine towards a given phenotype. Last, efforts on developing better SAM-based therapeutics should also address SAM's impermeability. More efforts could be directed towards developing SAM mimics that are more permeable to cell membranes or exploring MTA's therapeutic potential.

## Abbreviations:

AUC: – area under the curve,
MAT: – methionine adenosyltransferase,
MTA: – 5'-methylthioadenosine,
SAM: – S-adenosylmethionine.
